# Altered synaptic plasticity in the mossy fibre pathway of transgenic mice expressing mutant amyloid precursor protein

**DOI:** 10.1186/1756-6606-3-32

**Published:** 2010-11-01

**Authors:** Jonathan Witton, Jon T Brown, Matthew W Jones, Andrew D Randall

**Affiliations:** 1 School of Physiology and Pharmacology, University of Bristol, University Walk, Bristol, BS8 1TD, UK; 2 Pfizer Applied Neurophysiology Group, University of Bristol, University Walk, Bristol, BS8 1TD, UK

## Abstract

Aβ peptides derived from the cleavage of amyloid precursor protein are widely believed to play an important role in the pathophysiology of Alzheimer's disease. A common way to study the impact of these molecules on CNS function is to compare the physiology of transgenic mice that overproduce Aβ with non-transgenic animals. In the hippocampus, this approach has been frequently applied to the investigation of synaptic transmission and plasticity in the perforant and Schaffer collateral commissural pathways, the first and third components of the classical hippocampal trisynaptic circuit, respectively. Similar studies however have not been carried out on the remaining component of the trisynaptic circuit, the mossy fibre pathway. Using transverse hippocampal slices prepared from ~2 year old animals we have compared mossy fibre synaptic function in wild-type mice and their Tg2576 littermates which age-dependently overproduce Aβ. Input-output curves were not altered in slices from Tg2576 mice, but these animals exhibited a significant loss of the prominent frequency-facilitation expressed by the mossy fibre pathway. In addition to this change in short term synaptic plasticity, high frequency stimulation-induced, NMDA-receptor-independent LTP was absent in slices from the transgenic mice. These data represent the first description of functional deficits in the mossy fibre pathway of Aβ-overproducing transgenic mice.

## Background

The hippocampus is a brain structure with a crucial role in mammalian learning and memory. In transverse section the hippocampus has an iconic morphology around which a trisynaptic loop of glutamatergic pathways navigates. The three sequential glutamatergic pathways that make up this circuit are 1) the perforant path (PP) inputs to the granule cells of the dentate gyrus (DG-GC), 2) the mossy fibre projection (MFP) from DG-GCs to CA3 pyramidal cells (CA3-PC) and, 3) the Schaffer collateral commissural pathway (SCCP) that links CA3-PCs with CA1 pyramidal cells (CA1-PC).

There are a large number of neurological and psychiatric diseases in which measurable cognitive deficits contribute to the disease phenotype. Of these Alzheimer's disease (AD) is perhaps the best known. The prevalence of this condition means that most adults in the developed world will have personal experience from friends or family members of the devastating effects on cognitive function AD produces in those afflicted with the disease.

The majority of AD investigators would accept that Aβ peptides play a major role in the pathophysiology of AD [1,2]. These molecules are derived from proteolytic cleavage of amyloid precursor protein (APP) and APP mutations that favour Aβ generation or Aβ aggregation lead to familial forms of AD [3]. This has lead to an extensive, and ever-growing, literature on the biological actions of Aβ peptides. Large parts of this body of research involve two major experimental approaches. The first revolves around determining the effects produced by exogenous Aβ peptide preparations introduced acutely (or subacutely) into assay systems of interest. The second approach involves comparing the physiology of wild-type (WT) mice with examples of genetically engineered mouse lines that express transgenes which result in age-dependent over-production of Aβ [4]. There are now very large numbers (> 50) of such transgenic lines. The transgenes they express are usually APP variants that promote Aβ-production and/or aggregation, sometimes combined with other genes involved in the APP-processing pathway, most notably mutant forms of presenilin1 (PS1).

Numerous studies have considered how Aβ affects basal synaptic function and synaptic plasticity in the hippocampus [5]. These investigations have involved exogenous Aβ preparations of various forms as well as Aβ-overproducing transgenic animals. Interestingly, despite the large number of such studies performed in recent years, the effects of Aβ peptides have been only been investigated in two of the three pathways of the classic trisynaptic loop [5]. The pathways that have already been studied in depth, the PP and SCCP, are notably those that express NMDA-receptor dependent long-term potentiation (LTP). In contrast, the effects of Aβ on the MFP from DG granule cells to CA3 neurones, has received little if any attention.

As well as exhibiting presynaptically-expressed NMDA receptor-independent LTP, the MFP has a quite different physiology, anatomy and pharmacology to that of either the PP or SCCP [6-10]. Working in tandem with direct PP inputs to area CA3 the MFP is proposed to play a discrete role in hippocampal information processing and thus, by extension, hippocampally-mediated mnenomic function [11-14]. Here we present a first investigation of synaptic function in the MFP using hippocampal slices prepared from the commonly studied Aβ-over-expressing mouse line, Tg2576. When compared to WT littermates, we found both short and long term synaptic plasticity were altered in this transgenic line.

## Methods

The animals used for this study were 24-25 month old male Tg2576 mice and their WT littermates [15]. At this advanced age Tg2576 mice have both elevated Aβ and amyloid plaque pathology. Tg2576 mice express human APP harbouring the Swedish mutation (APPSwe) under control of the hamster prion protein promoter, which is expressed highly in the brain (but is not neuronal specific). The mice were on a mixed genetic background of C57Bl/6SJL and C57Bl/6. After weaning, selection of male pups and PCR-based genotyping the mice were singly housed on a 12:12 hr light:dark cycle with ad lib access to food and water.

Following sacrifice by cervical dislocation the hippocampus was dissected free from the rest of the brain and 400 μm thick transverse hippocampal slices were prepared using methods similar to those of others e.g. [16]. Following a post-slicing recovery period of > 1 hr slices were transferred to an interface-type recording chamber constantly perfused with artificial cerebrospinal fluid (aCSF) at 33°C. The composition of the aCSF was (mM) NaCl, 124; KCl, 3; NaHCO_3_, 26; CaCl_2_, 2; NaH_2_PO_4_, 1.25; MgSO_4_, 1; D-glucose, 10. This aCSF was equilibrated with 95% O_2 _and 5% CO_2 _by constant bubbling.

Extracellular recordings where made from *stratum lucidum *using aCSF filled glass electrodes of ~3 MOhm resistance. Field excitatory postsynaptic potentials (fEPSPs) were evoked in response to brief electrical stimuli applied via a concentric bipolar stimulating electrode placed deep in the hilar region close to the dentate granule cell layer. Responses were amplified (1000×), lowpass filtered at 10 kHz and digitally sampled to a personal computer at 50 kHz using pClamp10 software. All experiments were performed in the presence of L-689,560 (5 μM, Tocris) to entirely block NMDA receptor function. The experimenter was blind to genotype during the recordings.

**Figure 1:**
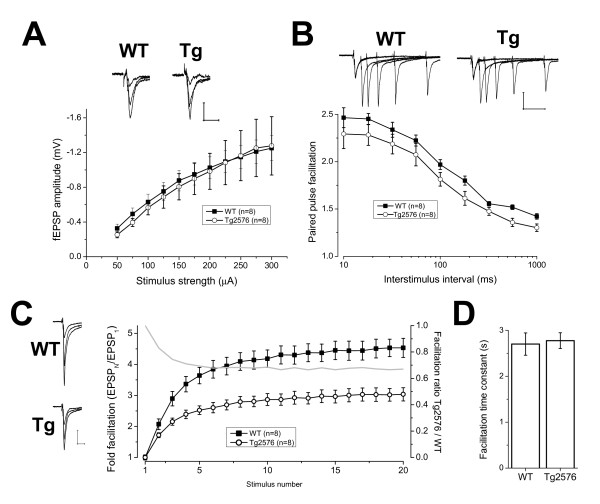
**Altered short term plasticity in the mossy fibre pathway of Tg2576 mice**. A) Pooled input-output curves from 8 WT and 8 Tg2576 recordings of mossy-fibre transmission. The graph plots applied stimulus strength versus EPSP amplitude. The inset traces illustrate overlaid responses to 50, 150 and 300 μA stimulation in the indicated genotype. Scale bar 10 ms, 0.5 mV. B) Pooled paired pulse facilitation data for inter-stimulus intervals from 10 to 1000 ms. The graph plots, for each inter-stimulus interval, the amplitude of the second evoked EPSP as a fraction of the first. The example recordings illustrate responses to paired stimuli with interstimulus intervals of 10-100 ms with a quarter log spacing. Data are from typical WT and Tg2567 recordings as indicated. Scale bar: 30 ms, 1 mV. C) The symbols plot, for WT and Tg2576 mice, the frequency facilitation profiles elicited by 20 pulse 1 Hz stimulus trains. The amplitude of the n^th ^EPSP of the train is plotted relative to the response to the 1^st ^stimulus. The grey line without symbols plots the ratio of the facilitation in Tg2576 slices to that in WT slices, and illustrates how the divergence grows rapidly over the first few stimuli. The traces to the left are overlays of the 1^st^, 3^rd ^and 20^th ^fEPSPs in example WT and Tg2576 recordings. Scale bar: 10 ms, 1 mV. D) A graph plotting the mean facilitation time constant for WT and Tg2576 recordings. These were derived by fitting single exponential functions to the frequency facilitation exhibited by each recording. The data reveal there was no difference in facilitation rate between WT and Tg2576 recordings. The units on the ordinate are stated in seconds but also could be quoted as number of stimuli. The data in B-D were obtained from 8 slices in each group, these were obtained from 5 WT and 7 Tg2576 mice, respectively.

Initially, input-output curves were collected by varying the stimulation strength applied in the range 50-300 μA, in 25 μA intervals. For subsequent investigations of synaptic plasticity the stimulus strength was set to a level that produced ~50% of the maximal response. As suggested by the pooled input-output data from the two genotypes (see Figure 1A), the stimulus intensity required to do this was not different in the two groups (WT 112.5 ± 4.7 μA, Tg2576 112.5 ± 9.4 μA).

Short-term synaptic plasticity was investigated in two ways. Firstly, we collected paired-pulse profiles with inter-stimulus intervals varying logarithmically over two orders of magnitude (10-1000 ms). Secondly, the increase in synaptic response seen during a 20 pulse, 1 Hz stimulus train was determined. To study LTP, we recorded baseline synaptic transmission in response stimuli applied every 30s for at least 10 minutes. We then attempted to induce NMDA receptor-independent LTP with a conditioning stimulus consisting of 1 s, 100 Hz stimulus train, repeated three times with an inter-train interval of 10 s. The responses to stimuli applied every 30s were then followed for another 30 minutes, at which time we determined the synaptic depression produced by application of DCG-IV (2 μM). Depression greater than 75% by this group II mGluR agonist was used to confirm we had been studying a largely pure MFP input [17].

Data were analysed using a combination of pClamp software and custom-written code within the MATLAB numerical processing environment. For LTP experiments amplitude measurements were made on averages of 4 consecutive EPSPs, each of which therefore cover a 2 minute time window. Short-term plasticity protocols were typically run three times on each slice and responses averaged. Input-output curves were compared with a 2-way ANOVA. Paired pulse facilitation at 1s was analysed using Student's t-test. This test was also used to compare the area under the frequency facilitation curves. Comparisons of the extent of LTP in WT and Tg2576 mice were based on a Student's t-test performed on the mean of the last 2 minutes of synaptic responses recorded before DCG-IV application.

## Results

MFP-mediated synaptic responses with classical fast negative-going waveforms and DCG-IV-sensitivity were present in hippocampal slices prepared from both WT and Tg2576 mice. Input-output curves were compiled with stimulus strengths between 50-300 μA. These exhibited the expected increase in EPSP amplitude as stimulus strength was increased. Although the relationship between EPSP amplitude and stimulus strength was, not unexpectedly, quite variable from slice to slice, pooled input-output curves from WT and Tg2576 mice were very similar (Figure 1A, P > 0.85). Thus, there does not seem to be a profound alteration to basal MF synaptic transmission in the Aβ-overproducing mice, or at least one that can be resolved with the extracellular recording methodologies employed here.

A hallmark of MF inputs to CA3-PCs in young animals is a pronounced short-term synaptic plasticity. This is evident in both substantial paired pulse facilitation (PPF) and frequency facilitation (FF) elicited by trains of stimuli. MFP responses in slices from both aged WT and Tg2576 mice exhibited marked PPF (Figure 1B); however, at all inter-stimulus latencies PPF in the Tg2576 group (n = 8) was between 5 and 10% less that than that in the WT group (n = 8), indicating that short term synaptic plasticity might be disturbed in the Aβ-overproducing mice. At the longest inter-stimulus interval tested, 1 s, PPF in Tg2576 slices was significantly smaller (91.6 ± 2.7%) than that in slices from WT mice (P < 0.03, unpaired t-test). When stimulation was repeatedly applied at this same inter-stimulus interval (i.e. 1 Hz) 20 times in succession marked frequency facilitation developed. Although facilitation occurred in both groups it was substantially attenuated in slices from Tg2576 mice (Figure 1C). The area under these facilitation curves was 57.4 ± 2.8 in WT slices and 33.3 ± 3.3 in Tg2576 mice, a highly significant difference (P < 0.00008, unpaired t-test). The rates with which frequency facilitation developed during these 1 Hz trains were not different, with time constants of 2.70 ± 0.24s and 2.78 ± 0.17s in WT and Tg2576 mice, respectively (P < 0.88, Figure 1D). As the number of stimuli delivered was increased, the ratio of the facilitation observed in WT and Tg2576 animals fell to a plateau of ~0.67 over the first ~6 stimuli (Figure 1C, grey line); this difference between the two groups developed exponentially with a rate of 0.76 stimuli^-1^.

**Figure 2:**
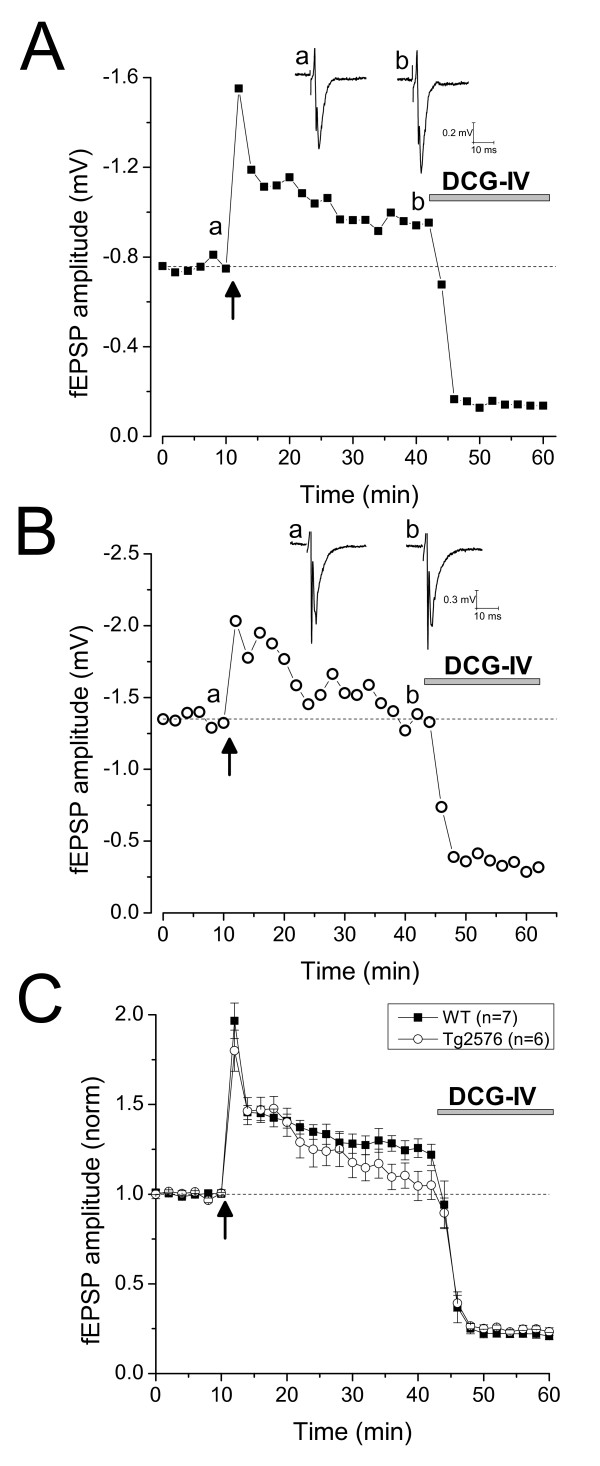
**Loss of NMDA-receptor independent LTP in the mossy fibre pathway of Tg2576 mice**. A) An example LTP experiment in a 25 month old WT mouse. After a 10 minute period of stable baseline EPSPs in response to low frequency stimulation (0.05 Hz), a conditioning stimulus consisting of 3, 1 s, 100 Hz train was applied at the time indicated by the arrow. Post conditioning low frequency stimulation-evoked EPSPs were followed for another 30 minutes after which time the synaptic depression produced by DCG-IV (2 μM) application was determined. The inset show example traces recorded just prior to the conditioning stimulus and just prior to DCG-IV application. B) An example recording similar to that in (A) but from a Tg2576 mouse. C) Pooled LTP data from 7 WT and 6 Tg2576 slices (taken from 4 and 5 animals, respectively). Note the LTP in slices prepared from Tg2576 mice does not persist. DCG-IV consistently depressed the synaptic responses by ~80% in both groups.

To investigate MF-LTP in these animals, we first recorded a period of stable baseline synaptic transmission in response to stimuli applied every 30 s. Following 10 or more minutes of stable EPSP amplitude we applied three 1s duration 100 Hz conditioning trains with an inter-train interval of 10 s. We then returned to stimulating synaptic responses every 30 s. As shown in both the example experiment (Figure 2A) and the pooled data set (Figure 2C), in WT animals the EPSP amplitude was approximately doubled in size in the 2 minutes immediately following application of the conditioning stimulus (1.97 ± 0.1 fold increase, n = 7). This post-tetanic potentiation rapidly decayed to a significant steady level of LTP which averaged 26 ± 5% greater than baseline 26-30 minutes after application of the conditioning stimulus (P < 0.01, n = 7, paired t-test). In slices from Tg2576 mice (Figure 2B and 2C) the post-tetanic potentiation observed immediately after the conditioning stimulus was 1.80 ± 0.11 fold (n = 6), not significantly different from that in WT mice (P > 0.29, unpaired t-test). However, after 30 minutes the EPSP amplitude had decayed to 1.05 ± 0.08 fold of baseline. This level was not significantly different from the EPSP amplitude prior to applying the conditioning stimulus (P > 0.44, paired t-test). Furthermore, comparison of the extent of LTP between Tg2576 and WT mice revealed a significant difference (P < 0.04, unpaired t-test).

To demonstrate that we were indeed studying MFP-mediated synaptic transmission the group II mGluR agonist DCG-IV was applied to every slice 30 minutes after the application of the 100 Hz conditioning stimuli [17]. As reported in younger animals, this agent produced a robust depression of the EPSP in both WT and Tg2576 mice (Figures 2A, B and 2C).

## Discussion

This manuscript provides a first description of alterations to MF synaptic function in a mouse model of AD-related Aβ accumulation. For this work we used a single cohort of Tg2576 mice which express the Swedish mutation of APP under the control of a prion protein promoter. These animals are an early example of the now numerous mouse lines genetically engineered to overproduce Aβ in an age-dependent fashion. Such animals are commonly used to experimentally model certain aspects of AD pathology [4] and are also widely employed in the quest to develop Aβ-lowering agents as potential therapies for AD.

Tg2576 mice were first produced some 15 years ago [15] and to date have been the most widely studied Aβ-overproducing mice. Tg2576 mice were the first to be used to demonstrate alterations to hippocampal synaptic function in both the PP input to the dentate gyrus and the SCCP input to area CA1 [18]. Numerous related studies have also been performed on more recently generated Aβ-overproducing lines, many of which exhibit a more aggressive and rapidly arising amyloidopathy [5]. These have included investigations of synaptic function and plasticity in both the PP and SCCP, which when compared reveal rather mixed and often conflicting findings [5].

Despite the numerous investigations in the PP and SCCP, to our knowledge there have been no previous studies of MF function in any Aβ overproducing mouse lines; although there has been a single study of the MFP in β-secretase knockout mice which are unable to produce Aβ. These β-secretase null animals exhibit enhanced MF paired-pulse facilitation and loss of LTP [19]. Here we describe how mossy fibre function is altered in Tg2576 mice. Our study was performed with mice aged ~2 years; thus, in addition to determining the consequences of an increased Aβ load at these advanced ages, our data also provide a first insight in of MF function in very aged WT mice.

2 year old WT mice were found to have extracellularly recorded synaptic responses that outwardly resemble the distinctive transient waveform of MF fEPSPs in younger animals. Our recordings also indicate that the presence of quite substantial, long-lasting (i.e. >1 s) paired-pulse facilitation (Figure 2B) combined with strong 1 Hz frequency facilitation (Figure 2C), both hallmarks of MF-CA3-PC transmission in younger animals [20], appear to persist into old age.

It has been previously shown that the extent of paired-pulse and frequency facilitation in the MFP of mice declines over the first 9 post natal weeks [21]. Although it is not easy to directly compare across laboratories due to differences in experimental methods (e.g. recording temperature, divalent ion concentration, species and strain employed), our data certainly indicate that there is not much further decline in the extent of MF short term plasticity as animals age from 9 weeks to 2 years, a supposition that is supported by other recent work on the MFP of adult rodents in our laboratory. It is also important to note that significant NMDA receptor-independent MF-LTP can also be evoked in aged WT mice; in our recordings it was seen in every slice, varying in amplitude from 7% to 45% of baseline amplitude, and was always preceded by a rapidly decaying post-tetanic potentiation.

In studies of the MFP it is standard practice to apply DCG-IV at the end of recordings. This is used to demonstrate that synaptic responses arise largely from stimulation of MFs, rather than recruitment of other glutamatergic pathways, notably the associational-commissural fibres, that can also generate glutamatergic synaptic responses in area CA3 [17]. We were reassured to see our synaptic responses were consistently depressed by around 80% by this group II mGluR agonist (Figure 2).

In contrast with studies of the SCCP in Aβ overproducing mice, including those performed in our laboratory [22-25], input-output properties of MF responses were not dependent on genotype (Figure 1A). In contrast, both short term synaptic plasticity and LTP in the MFP were modified in the Aβ peptide over-producing mice. The PPF deficit observed in Tg2576 was subtle (Figure 1B); for example, with two stimuli applied with a 1s inter-stimulus interval, PPF was ~8% smaller in Tg2576 mice. Although small, the PPF reduction in the Aβ-overproducing animals mirrors and indeed may be related to the enhanced PPF seen in BACE knockout mice, animals which can not produce Aβ [19]. Together these findings suggest Aβ may exert a tonic control of short term plasticity in wild-type non-transgenic mice. A similar conclusion was recently made from work largely based on hippocampal cultures [26]. Changes in PPF usually reflect an altered probability of release. The relatively minor (5-10%) decreases to PPF observed in the MFP of Tg2576 mice suggest that any underlying increases in release probability at stimulus-naïve synapses would also be small. We believe this explains why we do not see significantly enhanced input-output curves in the transgenic mice, especially in light of the considerable slice to slice variations in input-output relationships

Genotype-related differences in short-term MF plasticity were more markedly apparent in experiments in which 20 pulse, 1 Hz stimulus trains were used to induce frequency facilitation. The rate with which EPSPs in slices from Tg2576 mice increased was similar to that seen in WT mice (Figure 2D), but responses only tripled in size compared with a 4.5 fold change seen in WT animals. Interestingly, the changes in MF frequency facilitation in Tg2576 mice are very similar to those we have recently seen in TC1 mice at ~6 months of age. TC1 mice are a transchromosomic model of Down's syndrome, which like Tg2576 mice, exhibit deficits in hippocampal learning [27]. Unlike Tg2576 mice (Figure 2), however, TC1 mice do not have a parallel deficit in MF LTP (manuscript in preparation).

The marked frequency facilitation of normal MF responses is proposed endow this pathway with important circuit properties not seen in other hippocampal pathways. Thus, if individual DG cells fire repeatedly, the synaptic responses they generate in CA3 pyramids rapidly grow, such that one DG neurone can drive postsynaptic firing in CA3 cells [14]. Indeed, these features have lead to these synaptic connections being dubbed as "detonator synapses" [7]. Thus, the substantial loss of frequency facilitation in Tg2576 animals could have significant implications for how repetitive activity in DG-GCs is able to drive synaptic responses and subsequent action potential firing in post-synaptic CA3-PCs. In future, it will be interesting to examine the cellular basis for the depression of MF frequency facilitation. For example, an interesting potential avenue of investigation would be to examine if the presynaptic kainate receptor-mediated component of frequency facilitation [28] has been lost in MFs of Tg2576 mice.

It is also worth noting that both FF and PPF in MF are thought to reflect the actions of residual presynaptic Ca^2+^. Despite this common source, changes to the frequency facilitation appear to be more profound in Tg2576 mice. There are a number of potential causes for this that could be addressed in the future. For example, the difference between PPF and FF could arise from genotype-related differences in the dynamics or capacity of processes controlling presynaptic Ca^2+ ^homeostasis, or from differences in the size of the readily releasable vesicle pool.

In addition to changes to short-term plasticity, the ability of MF to exhibit activity-dependent LTP was compromised in 2 year old Tg2576 mice. Indeed, 30 mins after an induction protocol that induced LTP in all WT slices examined, there was no significant LTP in the Tg2576 group. The effects of transgenic Aβ overproduction on NMDA receptor-dependent LTP in the PP and SCCP remain somewhat controversial, with some laboratories seeing deficits and others, including ourselves, seeing no differences (reviewed by [5]). To some degree this research question is not aided by different groups using different transgenic lines, of different ages and different protocols. We believe that this is the first study of LTP in the MFP of an Aβ overproducing mouse, it will be interesting to see if other investigators make similar findings in future. Furthermore, a mechanistically different, NMDA receptor-dependent form of LTP has recently been described in the MFP of 2-4 week old rodents. This plasticity is postsynaptically-mediated and specifically involves potentiation of NMDA receptor-mediated synaptic responses [29,30]. Assuming this non-classical form MF LTP persists into adulthood, it would be informative to investigate if and how it is affected by Aβ accumulation.

In combination with alterations to other hippocampal circuits, the altered long- and short-term synaptic plasticity in the MF inputs to CA3-PC presumably contribute to altered hippocampal function that ultimately leads to learning and memory deficits in Aβ-overproducing mice, and by extension human AD sufferers. To fully understand all the consequences of prolonged Aβ accumulation for information transfer between DG and area CA3 it will also be important to investigate function and plasticity in MF connections with GABAergic interneurones. It will be particularly fascinating to examine the interneurones in stratum lucidum which are innervated by filopodial extensions from the large MF boutons that innervate pyramidal cells. Interestingly, at least in young animals, these connections often exhibit paired pulse depression and high frequency stimulation-induced long-term depression [31].

## Conclusions

We have provided the first evidence that MF transmission is disturbed in an Aβ-overproducing transgenic mouse line. This adds to the catalogue of neurophysiological deficits described in such models of AD-associated amyloidopathy, and uncovers the need for further AD-related investigations of this neurophysiologically unique component of the trisynaptic loop.

## Abbreviations

Aβ: amyloid beta peptide; AD: Alzheimer's disease; APP: amyloid precursor protein; DG: dentate gyrus; LTP: long-term potentiation; MF: mossy fibre(s); MFP: mossy fibre projection; PP: perforant pathway; PPF: paired pulse facilitation; SCCP: Schaffer collateral commissural pathway; WT: wild-type.

## Competing interests

The authors declare that they have no competing interests

## Authors' contributions

The experimental work was largely performed by JW with training and experimental and analytical support from JB. AR and MJ co-supervise JW and were involved in the planning of these studies, obtaining research funding and preparation of the manuscript. All authors read and approved the final manuscript.
